# Co-Localized in Amyloid Plaques Cathepsin B as a Source of Peptide Analogs Potential Drug Candidates for Alzheimer’s Disease

**DOI:** 10.3390/biom15010028

**Published:** 2024-12-30

**Authors:** Marilena K. Theodoropoulou, Konstantina D. Vraila, Nikos C. Papandreou, Georgia I. Nasi, Vassiliki A. Iconomidou

**Affiliations:** Section of Cell Biology and Biophysics, Department of Biology, School of Sciences, National and Kapodistrian University of Athens, Panepistimiopolis, 157 01 Athens, Greece; mtheod@biol.uoa.gr (M.K.T.); vrailakon@biol.uoa.gr (K.D.V.); npapand@biol.uoa.gr (N.C.P.); gnasi@biol.uoa.gr (G.I.N.)

**Keywords:** Alzheimer’s disease, Cathepsin B, aggregation-prone regions, amyloid-*β*, A*β*, molecular dynamics, umbrella sampling

## Abstract

Alzheimer’s disease (AD) is a complex neurodegenerative disorder characterized by extracellular amyloid plaques, predominantly consisting of amyloid-*β* (A*β*) peptides. The oligomeric form of A*β* is acknowledged as the most neurotoxic, propelling the pathological progression of AD. Interestingly, besides A*β*, other proteins are co-localized within amyloid plaques. Peptide analogs corresponding to the “aggregation-prone” regions (APRs) of these proteins could exhibit high-affinity binding to A*β* and significant inhibitory potential against the A*β* oligomerization process. The peptide analogs of co-localized protease, Cathepsin B, may act as such potent inhibitors. In silico studies on the complexes of the oligomeric state of A*β* and Cathepsin B peptide analogs were performed utilizing molecular docking and molecular dynamics simulations, revealing that these analogs disrupt the *β*-sheet-rich core of A*β* oligomers, a critical structural feature of their stability. Of the four peptide analogs evaluated, two demonstrated considerable potential by effectively destabilizing oligomers while maintaining low self-aggregation propensity, i.e., a crucial consideration for therapeutic safety. These findings point out the potential of APR-derived peptide analogs from co-localized proteins as innovative agents against AD, paving the way for further exploration in peptide-based therapeutic development.

## 1. Introduction

Alzheimer’s disease (AD) is a multifactorial neurodegenerative disorder and the leading cause of dementia worldwide. It is characterized by the progressive loss of brain functions such as memory, thinking and behavior due to the neurons’ degeneration in key cortical areas [1]. One of the major pathological hallmarks of AD is the presence of extracellular amyloid plaques, which are mainly composed of amyloid-β (A*β*) peptide. A*β* is the proteolytic product of amyloid precursor protein (APP), a transmembrane protein involved in synapse formation, the defense mechanisms of neuronal cells and transmembrane transport of iron ions. During the amyloidogenic proteolytic pathway, β-secretase and γ-secretase sequentially cleave APP, producing A*β* peptides of varying lengths, with A*β*_40_ and A*β*_42_ being the most abundant forms in the brain [2]. The accumulation of A*β* peptides can lead to the formation of amyloid fibrils, a nucleation-dependent polymerization process, where monomeric A*β* peptides form oligomers that subsequently interact to form the thermodynamically preferred fibrils with the characteristic cross-β structure [3]. The elimination of A*β* aggregates can delay the formation of amyloid fibrils and may offer therapeutic potential for AD [4].

While strategies focused on reducing amyloid plaque size have not significantly delayed disease progression and amyloid plaque formation [5], inhibiting Aβ peptide accumulation using antibodies has shown promise, leading to behavioral improvements in transgenic mice [6]. As a result, new therapeutic strategies focus on either inhibiting the A*β* oligomerization process and interfering with A*β* accumulation, or destabilizing existing A*β* oligomers [7]. Recent studies have employed virtual screening, molecular dynamics (MD) simulations, in vitro experiments and clinical trials to target the soluble, toxic seed of the amyloidogenic process [8]. These approaches hold promise for identifying novel therapeutics for the treatment of Alzheimer’s disease.

Our study focuses on identifying potential peptide drugs that exhibit a high affinity for A*β* and inhibit its oligomerization. Peptide-based drugs are considered a promising therapeutic option for AD due to their ability to cross the blood–brain barrier (BBB) [9] and their favorable pharmacokinetic properties, including efficient hepatic and renal clearance [10]. Unlike small molecules, peptides offer advantages, such as a larger surface area, greater structural complexity, and the ability to be synthesized rapidly and reliably. Several peptide-based drugs, such as insulin for diabetes and enfuvirtide for HIV, have already been approved, demonstrating their therapeutic potential [11].

A promising source for developing candidate peptide drugs against AD is the co-localized proteins on A*β* fibrillar deposits. Besides the main amyloid fibril protein, amyloid deposits contain co-localized proteins known as amyloid signature proteins (ISA, 2024) [12]. While the precise role of these proteins in the oligomerization and amyloid formation is still not fully understood, it is known that some of them can form amyloid fibrils or have the potential to partially unfold and form amyloid fibrils under certain conditions [13]. Thus, these proteins contain regions known as “aggregation-prone” regions (APRs), which share the same physicochemical properties as the APR ^16^KLVFF^20^ of A*β*. APRs are short segments that regulate the self-aggregation and subsequent misfolding into amyloid-like structures [14]. Peptide analogs corresponding to Aβ-co-localized proteins could therefore have a strong binding affinity to A*β*, leading to the design of peptide drugs that specifically interact with A*β* oligomers and disrupt the amyloidogenic process. For instance, recent work by our group suggests that peptide analogs derived from Clusterin, a protein co-localized with Aβ, can inhibit fibril formation. These peptides analogs correspond to APRs that are located in the α-chain of Clusterin [15].

Cathepsin B, another co-localized protein, is a cysteine protease involved in endolysosomal activity. It has been shown that Cathepsin B participates in the cleavage of APP and the production of A*β* by exhibiting β-secretase activity [16]. Based on that observation, Cathepsin B inhibitors have been tested for the reduced production of *Aβ* and may constitute candidate drugs for AD [16,17]. On the contrary, Mueller-Steiner et al. have suggested that Cathepsin B reduces amyloid plaque deposition and relative A*β*_42_ levels [18].

Our study was propelled by the pivotal role of A*β* oligomers in the progress of AD, and the distinct therapeutic benefits of peptide-based drugs, especially their ability to precisely target intricate protein interactions, such as those observed in AD [19]. Unlike previous studies that focus solely on the inhibition of A*β* production or plaque formation, we explored a novel approach by identifying and evaluating peptide analogs generated from the APRs of Cathepsin B, a protease co-localized within amyloid plaques. Utilizing molecular docking and molecular dynamics (MD) simulations, we demonstrated the capability of these peptide analogs to destabilize A*β* oligomers by targeting their pathological *β*-sheet-rich conformation. Furthermore, by examining their self-aggregation propensity, we established a vital assessment criterion to ensure both effectiveness and safety. This study presents an intriguing framework for employing co-localized protease-derived peptides as dual-purpose agents: destabilizing toxic A*β* oligomers while minimizing the risk of aggregation, hence facilitating the development of revolutionary peptide-based therapies for AD.

## 2. Materials and Methods

### 2.1. Modeling Structures of Aβ_42_ Oligomers

The NMR-derived three-dimensional (3D) structure of the A*β*_42_ oligomer was retrieved from the Protein Data Bank (PDB ID: 2BEG). This entry contains ten conformers with the least restraint violations, and each conformer consists of five chains [8] which are labeled A, B, C, D and E respectively. Model 9, which is the overall representative medoid model (most similar to other models), was used in our in silico experiments. This pentameric structure was selected due to the fact that it corresponds to the smallest detectable oligomer in solution [20], and will henceforth be referred to in our work as the A*β* oligomer. Although the N-terminal residues 1–16 of A*β*_42_ are absent in this model as they are disordered, it has been hypothesized that they do not contribute to the cross-β structure or the fibril stability. Both in vitro [21] and in silico [22] experiments indicate that these residues mostly form a region with low hydrophobicity and do not participate in the formation of fibrils. The remaining residues (18–42) adopt a *β*-strand–turn–*β*-strand motif that contains two intermolecular, parallel, in-register *β*-sheets.

### 2.2. Modeling the Structures of Peptide Analogs

The sequence of Cathepsin B (Uniprot AC: P07858 [23]) was retrieved from AmyCo, a publicly available collection of amyloidoses and other clinical disorders related to amyloid deposition [24]. AmyCo provides, among other data, information on both the amyloidogenic and co-deposited proteins found in each disease. To identify the potential APRs of Cathepsin B and design the respective peptide analogs, we utilized the consensus algorithm AMYLRED2 [25]. However, since Cathepsin B participates as a β-secretase in APP cleavage, peptides corresponding to the catalytic site of Cathepsin B are not considered potential peptide drugs [26]. Thus, four peptide analogs were designed, two of them corresponding to the predicted APRs. Also, the extended sequence of each APR was selected and studied. The chosen peptides are listed in Table 1.

Peptide analogs CathB1a and CathB2a (sequence length 6 and 8 residues, respectively) were built utilizing PyMOL software package version 2.4 [27]. The structures of peptide analogs CathB1 and CathB2 (sequence length 21 and 18 residues, respectively) were isolated from the experimentally determined structure of Cathepsin B (PDB ID: 6AY2) [28].

### 2.3. Molecular Docking Experiments

Each peptide analog was docked to the A*β*_42_ oligomer by utilizing the automated protein docking server ClusPro [29,30,31,32]. Additionally, in order to study the potential of the peptide analogs to self-assemble, a complex consisting of five copies of each peptide was built utilizing the multimer-docking routine of ClusPro.

ClusPro was utilized for the generation of the peptide–Aβ complexes by applying the default settings of the tool. Its search algorithm (Piper) outputs the 1000 lowest-energy docked structures to the clustering algorithm. The final 10 proposed models represent the central structure of the clusters containing members with a 9 Å C-alpha RMSD radius. Ranking is performed according to the size of each cluster and then according to the lowest energy based on the balanced scoring function. So, after the use of the CluPro web server, relying on its successful predictions in CAPRI Challenge [32], the top ranked conformation for each complex was selected for the subsequent molecular dynamics simulations.

### 2.4. Molecular Dynamics Simulations

Next, the derived complexes were subjected to molecular dynamics (MD) simulations via the GROMACS software package (University of Groningen, Groningen, The Netherlands) version 2018.1 [33,34].

Each system was placed in a 1.2 nm size cubic box of the 3-point model (TIP3P) water [35]. For the simulations, the AMBER99SB-ILDN protein, nucleic AMBER94 force field was employed [36], as it has been shown to be more accurate for modeling disordered peptides by combining enhanced-sampling MD simulations with NMR data [37] and good at reproducing NMR observables for Alzheimer’s A*β*_42_ peptide [38]. For the achievement of neutral conditions, five sodium ions had to be added to each system.

Afterward, energy minimization was completed in a maximum of 2000 steps using the steepest descents algorithm. Then, two equilibration steps were performed with position restraints applied to heavy atoms throughout. The first equilibration step was carried out under NVT conditions at 310 K using coupling to the temperature bath (Berendsen thermostat) [39]). The second equilibration step was carried out under NPT conditions and the pressure was isotopically controlled at 1.013 bar (1 atm) using a barostat (Berendsen weak bath) [39] and Berendsen thermostat at 310 K. All the equilibrations were performed for 100 ps (50,000 steps of 2 fs). Finally, the MD simulation with position restraints removed was performed for 500 ns at 310 K for the complexes between each peptide analog and A*β*. The periodic boundary conditions were applied in all directions. The constraint algorithm used for bond lengths was LINCS (LINear Constraint Solver) [40]. The neighbor search cut-off was at approximately 0.8 nm. For the calculation of long-range electrostatic interactions, the fast smooth Particle Mesh Ewald summation method was used, while the Fourier grid spacing was set at 0.12 nm [41].

The simulations were conducted on the National HPC facility ARIS, managed by the Greek Research and Technology Network (GRNET). A total of 200 CPU cores were utilized, distributed across 10 nodes with 20 tasks per node, with each task corresponding to a single CPU core. The memory allocation was set to 1024 MB per CPU core, resulting in a total of 200 GB for the job. The wall-clock time for the simulations was limited to 48 h per job. GROMACS version 2018.1 was employed for molecular dynamics simulations, leveraging the IntelMPI library for efficient parallel processing. All computations were performed within the “compute” partition of ARIS, under project ID PR007003-AbetaDynamics.

Finally, the analysis of the complexes was performed utilizing built-in tools of the GROMACS software package version 2018.1. Structural stability was inspected using the rms tool [42]. The number of residues that are assigned to various secondary structural elements was calculated using the do_dssp tool [43,44]. The number of hydrogen bonds between the oligomer and the peptide or between the chains of the oligomer or between the residues on either side of the turn was calculated using the hbond tool [45]. The root mean square fluctuation (RMSF) was also calculated as a metric of individual residue flexibility, utilizing the GROMACS module rmsf. Last but not least, the compactness of the structure of the A*β*_42_ oligomer was evaluated by calculating the radius of gyration (Rg) with the use of the GROMACS module gyrate. Snapshots from the production run, extracted every 100 ps, were used for the analysis.

Self-assembly MD simulations were performed by following the same procedure described above for a total time of 1000 ns. Pictures were collected with the molecular visualization system UCSF Chimera [46].

### 2.5. Umbrella Sampling Method

The relative free binding energy of the complexes was calculated via the umbrella sampling method [47] that is also provided by GROMACS. The method was applied for the calculation of the energy that is required to pull away each peptide from the A*β* oligomer. Each system was placed in a box with dimensions 6.5 nm, 6.5 nm, and 15 nm, and energy minimization and equilibration processes were performed as described above. Afterward, an MD simulation of the peptide being pulled away from the A*β* oligomer was performed for 1 ns. The coordinates of the system were saved every 2 ps and certain frames were selected among a set of 500 conformers based on the distance (0.1 nm) between the atoms of the system of each frame.

Finally, for each selected frame, MD simulations were performed for 10 ns and the results were used for WHAM (weighted histogram analysis method) analysis and PMF (potential of mean force) calculations that revealed the strength of the interactions between the A*β* oligomer and each peptide analog.

**Figure 1:**
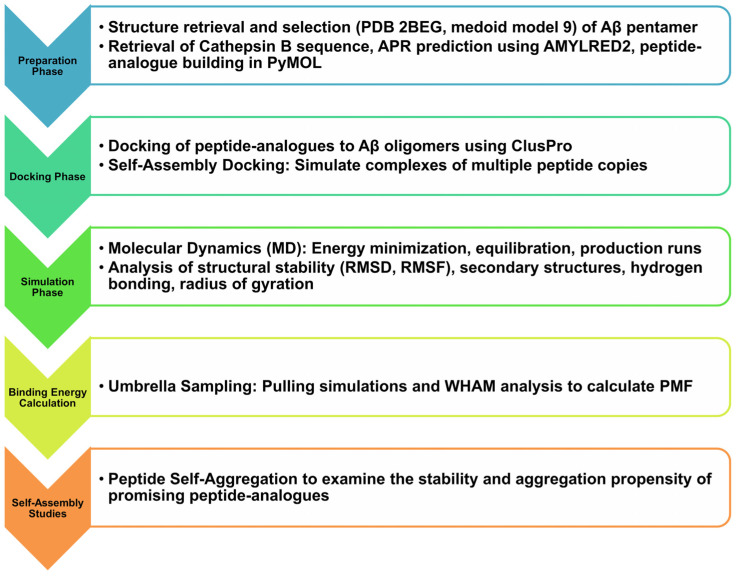
Graphical representation of the methods employed in the present study, alongside the materials and programs deployed.

The research workflow employed in this study, alongside the methods and software utilized, is presented in Figure 1.

## 3. Results

**Figure 2:**
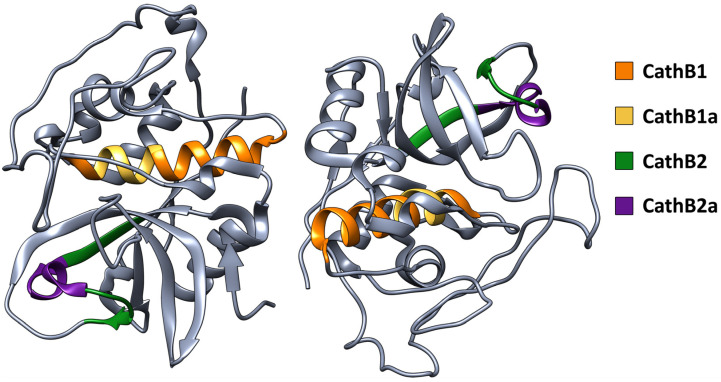
Graphical representation of the predicted “aggregation-prone” regions on the functional dimer of Cathepsin B. The CathB1 peptide analog is colored orange, the CathB1a peptide analog is yellow, the CathB2 peptide analog is forest green, and the CathB2a peptide analog is purple.

The peptide analogs that were selected to be studied as promising peptide drugs corresponded to the “aggregation-prone” regions of Cathepsin B (Figure 2).

### 3.1. Effect of the Peptide Analogs of Cathepsin B on Aβ Oligomer Structure

In order to study the effect of these four peptide analogs (listed in Table 1) on the oligomeric state of A*β,* we conducted MD simulations for 500 ns. The complexes of each peptide with the A*β* oligomer were analyzed throughout the simulation by creating various informative diagrams. These include the root mean square deviation (RMSD diagram) for the structure of the A*β* oligomer, the number of residues of the A*β* oligomer that participate in certain secondary structural elements (secondary structure diagram), the root mean square fluctuation (RMSF diagram) that represents the flexibility of the residues forming the A*β* oligomer, and the number of hydrogen bonds that are formed between the pairs of the A*β* chains and stabilize its oligomeric state. In addition, we examined the radius of gyration (Rg) plots, which provide insight into the compactness of the A*β* oligomer, to monitor alterations in its structural dispersion over time. Moreover, we calculated the average number of hydrogen bonds formed between the chains of A*β* and the average number of residues that are assigned to *β*-strands (that is the main secondary structural element of the A*β* oligomer) during the first 10 ns and last 10 ns of the simulation.

**Figure 3:**
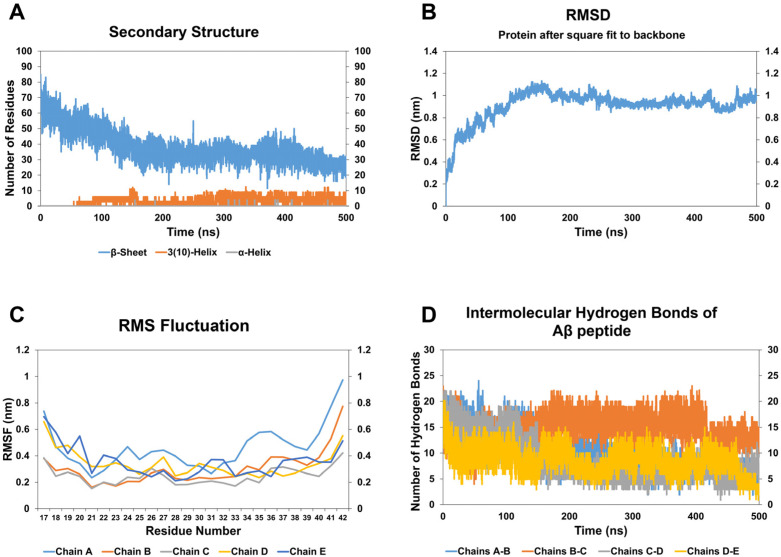
Results of molecular dynamic simulations of A*β*_42_ oligomer with the CathB1 peptide analog. (**A**) The number of residues assigned to the beta-sheet (blue), 3(10)-helix (orange), and a-helix (grey) of the A*β*_42_ oligomer during the simulation, according to DSSP calculations. (**B**) The RMSD of the A*β*_42_ oligomer during the course of the simulation. (**C**) The RMSF calculation for the residues of chain A (light blue), chain B (orange), chain C (grey), chain D (yellow), and chain E (dark blue) of the A*β*_42_ oligomer. (**D**) The number of hydrogen bonds formed between the chains of the A*β*_42_ oligomer and more particularly between chains A and B (blue), chains B and C (orange), chains C and D (grey), and chains D and E (yellow).

In the case of the A*β* oligomer-CathB1 peptide complex, the number of residues participating in *β*-strands significantly decreased by 52.67%, as calculated by the average count of *β*-strand residues during the initial and final 10 ns of the simulation (Figure 3A, Table 2). The decrease in *β*-strand content indicates the disruption of the oligomer’s cross-*β*-sheet motif, a secondary structural characteristic essential for its stability and aggregation capacity. The RMSD analysis (Figure 3B) revealed substantial structural modifications in the oligomer throughout the simulation, with an apparent increase after 50 ns, correlating with the observed reduction in *β*-strands. The visual inspection of simulation frames (Supplementary Figure S1) corroborates this observation, suggesting a gradual loss of *β*-strands and the development of disordered regions over time. Concurrently, the emergence of helical structural components, noted around 50 ns and sustained until the end of the simulation, signifies a transition toward less ordered, destabilized conformations (Figure 3A). The transitions are visually apparent in subsequent frames, when the peptide’s binding seems to prompt the reorganization of secondary structural elements (Supplementary Figure S1). The results of the RMSF diagram (Figure 3C) demonstrated increased flexibility regarding the residues that correspond to the five chains of A*β* oligomer, especially in the intrinsically dynamic N-terminal and C-terminal regions of A*β*, as well as at residue 42 within the *β*-strand–turn–*β*-strand motif. This increase compromises the structural rigidity of the oligomer, diminishing its capacity to preserve its compact *β*-sheet-rich architecture. Furthermore, a 50.66% decrease in inter-chain hydrogen bonding, essential for stabilizing the *β*-sheet core, was observed (Figure 3D, Table 2), further contributing to the oligomer’s destabilization. The peptide-induced structural changes and increased flexibility align with the radius of gyration (Rg) findings, indicating a general expansion of the oligomer structure and a reduction in compactness (Supplementary Figure S5A). The frames distinctly depict this structural expansion over time, illustrating the dynamic effects of CathB1 binding.

**Figure 4:**
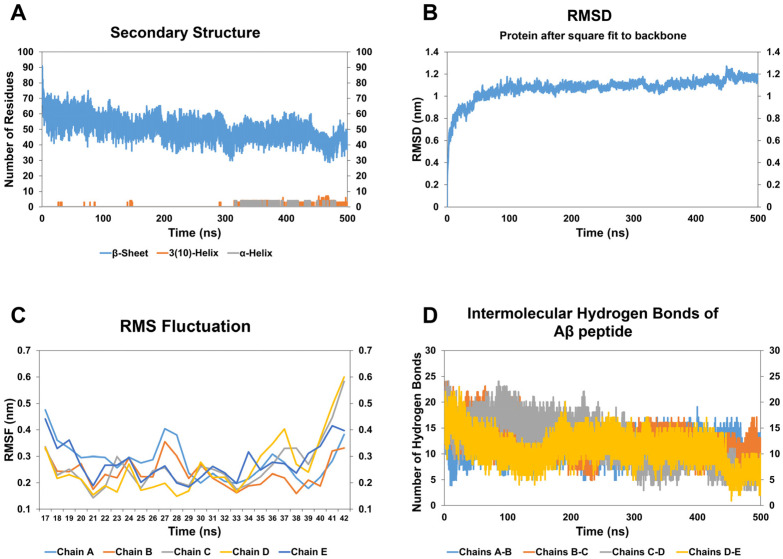
The results of molecular dynamic simulations of A*β*_42_ oligomer with CathB2 peptide analog. (**A**) The number of residues assigned to beta-sheet (blue), 3(10)-helix (orange), and a-helix (grey) of the A*β*_42_ oligomer during the simulation, according to DSSP calculations. (**B**) The RMSD of the A*β*_42_ oligomer during the course of the simulation. (**C**) The RMSF calculation for the residues of chain A (light blue), chain B (orange), chain C (grey), chain D (yellow), and chain E (blue) of the A*β*_42_ oligomer. (**D**) The number of hydrogen bonds formed between the chains of A*β*_42_ oligomer and, more particularly, between chains A and B (blue), chains B and C (orange), chains C and D (grey), chains D and E (yellow).

The effect of CathB2 peptide on A*β* oligomer is relatively moderate compared to CathB1 peptide. Specifically, the RMSD analysis (Figure 4B) demonstrated an upward trend in structural deviation during the simulation, indicative of moderate conformational alterations induced by the interaction of the oligomer with the peptide analog CathB2. The RMSD trajectory remained relatively stable after 320 ns, implying that structural transitions, including the formation of helical elements, achieved equilibrium by this time. This aligns with secondary structure analysis, which showed a 33.61% decrease in the number of residues involved in *β*-strands throughout the simulation (Figure 4A, Table 2). This reduction suggests a partial disruption of the *β*-sheet structure, crucial for the stability of the oligomer of A*β*. Upon visual examination of the respective simulation frames (Supplementary Figure S2), a progressive loss of *β*-strands over time was observed, accompanied by the appearance of increasingly disordered regions and new structural features. At 320 ns, the formation of 3_10_ helices became evident, persisting until the end of the simulation (Figure 4A). The helices, visible in the latter frames, further underline the transition from a stable *β*-sheet-rich configuration to a less ordered, flexible state. By analyzing the RMSF plots (Figure 4C), heightened flexibility in the C-terminal regions of all chains is highlighted, with chains C and D displaying the most significant fluctuations. This localized increase in flexibility corresponds with the structural changes identified in the secondary structure analysis and the disruption of inter-chain hydrogen bonds. In regards to hydrogen bond analysis (Figure 4D), a 23.30% decrease in inter-chain hydrogen bonds was identified (Table 2), signifying partial instability of the oligomer’s *β*-sheet core. The decline was most noticeable in interactions involving chains C and D, as demonstrated in the simulation frames, where a loss of compactness and increased dynamics in these regions were obvious. Moreover, the Rg plots (Supplementary Figure S5C) indicated a moderate increase in structural dispersion, consistent with the observed decrease in compactness. Collectively, the data point out that the peptide analog CathB2 destabilizes the structure of the A*β* oligomer by disrupting *β*-strands, enhancing residue flexibility, and weakening inter-chain hydrogen bonds. While its destabilizing effects are less pronounced than those of the peptide analog CathB1, CathB2 still induces localized structural alterations that contribute to the oligomer’s partial disintegration.

The peptide analogs CathB1a and CathB2a induce substantial conformational changes, akin to those observed with CathB1, as indicated by the respective MD simulations.

**Figure 5:**
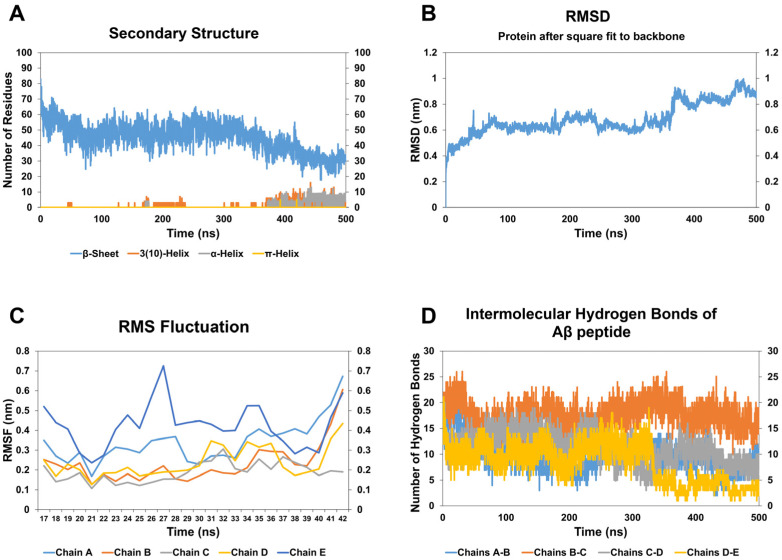
The results of molecular dynamic simulations of A*β*_42_ oligomer with CathB1a peptide analog. (**A**) The number of residues assigned to beta-sheet (blue), 3(10)-helix (orange), and a-helix (grey) of the A*β*_42_ oligomer during the simulation, according to DSSP calculations. (**B**) The RMSD of the A*β*_42_ oligomer during the course of the simulation. (**C**) The RMSF calculation for the residues of chain A (light blue), chain B (orange), chain C (grey), chain D (yellow), and chain E (blue) of the A*β*_42_ oligomer. (**D**) The number of hydrogen bonds formed between the chains of A*β*_42_ oligomer and more particularly between chains A and B (blue), chains B and C (orange), chains C and D (grey), and chains D and E (yellow).

In the A*β* oligomer–CathB1a peptide complex, the number of residues that are assigned to *β*-strands decreases significantly by 47.92% (Figure 5A, Table 2). This reduction reflects a significant disruption of the oligomer’s cross-*β*-sheet motif. The formation of α-helices in the final 150 ns of the simulation, especially in chain E, underscores a transition from *β*-sheet configurations to less compact and ordered conformations (Figure 5A). These transitions are also evident in the simulation frames (Supplementary Figure S3), which illustrate the gradual reduction of *β*-strands and the emergence of helical regions over time. While examining the RMSD diagram (Figure 5B), a steady increase in structural deviations is revealed over the course of the simulation, particularly post 200 ns, suggesting considerable peptide-induced conformational alterations in the oligomer. These modifications coincide with the RMSF data (Figure 5C), indicating enhanced flexibility in the C-terminal regions of chains A and B, as well as in chain E, especially in residues associated with the turn of the *β*-strand-turn-*β*-strand motif. Increased flexibility in these regions compromises the oligomer’s structural integrity and diminishes its capacity to sustain a compact *β*-sheet-rich conformation. A significant decrease in the number of hydrogen bonds formed between the chains of A*β* oligomer was also detected (Figure 5D), estimated at 42.82% (Table 2), hence exacerbating instability. This reduction disrupts essential interactions that stabilize the oligomer core, with the loss of hydrogen bonds being most noticeable in the regions associated with chains D and E, as observed in the simulation frames. Additionally, the Rg analysis (Supplementary Figure S5B) demonstrated a significant increase in structural disruption throughout the simulation, revealing reduced oligomer compactness. The frames further emphasize this expansion, exhibiting a discernible loosening of the oligomer structure and a clear reduction in *β*-sheet alignment. Hence, these observations highlight the ability of peptide analog CathB1a to destabilize the A*β* oligomer by disrupting its secondary structural elements and weakening its compactness, effectively hindering its potential to initiate amyloid fibril formation.

**Figure 6:**
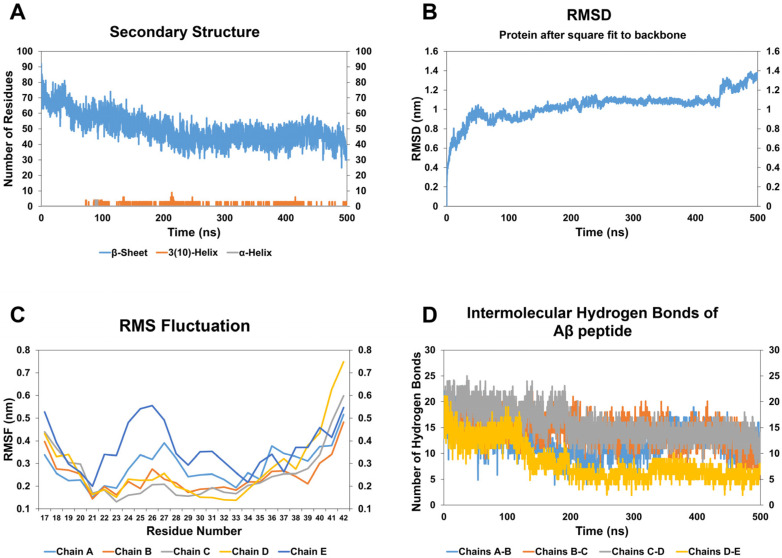
The results of molecular dynamic simulations of A*β*_42_ oligomer with CathB2a peptide analog. (**A**) The number of residues assigned to beta-sheet (blue), 3(10)-helix (orange), and a-helix (grey) of the A*β*_42_ oligomer during the simulation, according to DSSP calculations. (**B**) The RMSD of the A*β*_42_ oligomer during the course of the simulation. (**C**) The RMSF calculation for the residues of chain A (light blue), chain B (orange), chain C (grey), chain D (yellow), and chain E (blue) of the A*β*_42_ oligomer. (**D**) The number of hydrogen bonds formed between the chains of A*β*_42_ oligomer and more particularly between chains A and B (blue), chains B and C (orange), chains C and D (grey), and chains D and E (yellow).

Finally, in the case of the A*β* oligomer–CathB2a peptide complex, a notable decrease in the number of residues designated as *β*-strands was recorded, estimated at 44.91% (Figure 6A, Table 2). This reduction reflects a considerable disruption of the oligomer’s *β*-sheet architecture, critical for its structural stability and aggregation potential. The visual inspection of the simulation frames (Supplementary Figure S4) corroborates this finding, showing a progressive loss of *β*-strands and the development of disordered regions over time. Remarkably, a stable 3_10_ helix was formed early in the simulation and maintained throughout, indicating a localized rupture of the *β*-sheet motif and a transition to less compact conformations (Figure 6A). The RMSD analysis (Figure 6B) exhibited an upward trend in structural deviation throughout the simulation, stabilizing after approximately 250 ns. This pattern aligns with the structural transitions observed in the secondary structural analysis, suggesting that peptide binding induces consistent conformational alterations in the oligomer. Residue flexibility, as seen by RMSF plots (Figure 6C), was particularly accentuated in the C-terminal regions of all five chains and in chain E at the *β*-strand–turn–*β*-strand motif. This increase in flexibility, also evident in the simulation frames, highlights the localized destabilization caused by the peptide analog CathB2a. Furthermore, the examination of hydrogen bonding patterns (Figure 6D) revealed a 46.98% decrease in the number of hydrogen bonds formed between the chains of A*β* oligomer (Table 2), especially in interactions between chains D and E. This disruption of essential stabilizing connections further exacerbates the destabilization of the oligomer. The analysis of the Rg plots (Supplementary Figure S5D) demonstrated a continuous increase in structural dispersion, denoting reduced compactness and increased oligomer flexibility. The frames visually represent this expansion, illustrating the gradual disintegration of the oligomeric structure. Overall, these results indicate that the peptide analog CathB2a proficiently propels the A*β* oligomer into a destabilized, non-aggregating state, by reducing *β*-strand content, disrupting hydrogen bonds, and enhancing flexibility in critical regions.

### 3.2. Relative Free Binding Energy Calculations of the Complexes by the Umbrella Sampling Method

**Figure 7:**
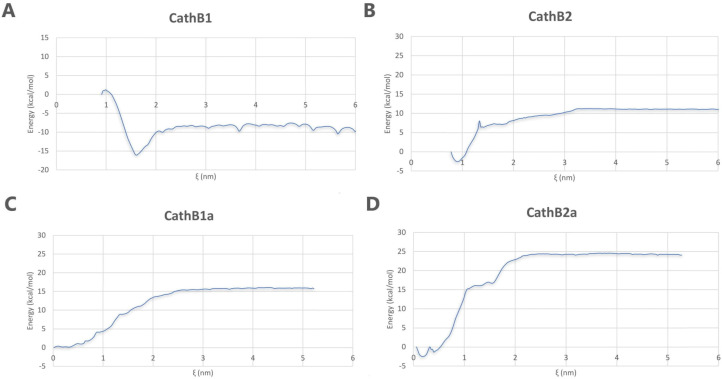
PMF profiles for peptide–A*β* oligomer complexes. Subfigures (**A**–**D**) depict the energy fluctuations (kcal/mol) relative to the reaction coordinate (ξ) for peptide analogs CathB1 (**A**), CathB2 (**B**), CathB1a (**C**), and CathB2a (**D**).

The umbrella sampling method was employed to quantitatively evaluate the effect of the examined peptide analogs on the destabilization of the A*β* oligomer through calculating the relative binding free energies of the peptide–A*β* oligomer complexes. WHAM analysis for all complexes was utilized to produce the potential of mean force (PMF) profiles, depicting the energy landscape along the reaction coordinate (Figure 7).

The results of WHAM analysis demonstrated unique binding energy properties among the peptide analogs. Notably, peptide analogs CathB1a and CathB2a exhibited the most stable interactions with the oligomer of A*β*, since the free energy values (ΔG) were estimated at roughly –15 kcal/mol and –25 kcal/mol, respectively (Figure 7C,D). Peptide analogs CathB1 and CathB2 presented comparatively fewer stable interactions, as evidenced by the elevated energy values in their respective PMF profiles (Figure 7A,B).

The specific findings, along with the structural analysis presented in Section 3.1, identify peptide analogs CathB1a and CathB2a as the most promising candidates, and were further studied for their possible destabilizing effects on A*β* oligomers.

### 3.3. Self-Assembly Studies of Peptide Analogs CathB1a and CathB2a

**Figure 8:**
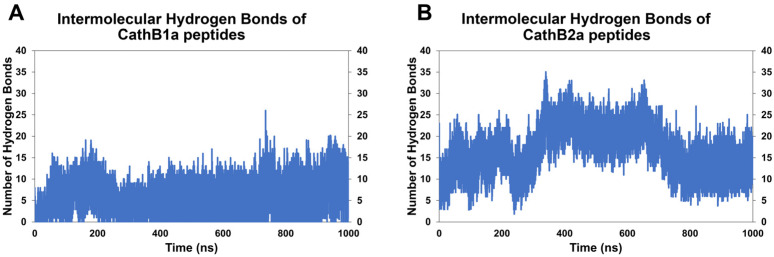
MD simulations of intermolecular interactions between CathB1a and CathB2a peptide analogs. The number of hydrogen bonds formed between the peptide analogs were calculated: (**A**) CathB1a; (**B**) CathB2a.

The fact that peptide analogs CathB1a and CathB2a show the most promising results led us to investigate computationally if these peptides have the potential to self-aggregate and form amyloid fibrils. For that purpose, five copies of each peptide analog were docked utilizing the multimer-docking routine of ClusPro, and self-assembly MD simulations were performed for 1000 ns. The presence of intermolecular hydrogen bonds is a crucial indication of the peptides’ capacity to form stable aggregates. A higher number of stable hydrogen bonds over the simulation period showcases a significant likelihood of self-aggregation and possible amyloid fibril formation. Consequently, to ascertain whether each peptide analog has a propensity for self-aggregation, diagrams demonstrating the intermolecular hydrogen bonds formed throughout the simulation were generated (Figure 8). Additionally, structural snapshots of the peptide analogs at designated time intervals (0 ns, 200 ns, 400 ns, 600 ns, 800 ns, and 1000 ns) were produced to visualize their conformational alternations over time (Supplementary Figures S6 and S8). The secondary structural modification of peptide analogs CathB1a and CathB2a throughout the simulation was meticulously examined, and diagrams depicting the beta-sheet and helix content over time were created for both peptide analogs (Supplementary Figures S7 and S9, respectively).

According to the results, CathB1a peptide analogs were detected to form stable intermolecular hydrogen bonds, with a consistent beta-sheet content observed throughout the simulation (Figure 8A). The specific observations demonstrate an apparent propensity for self-aggregation, as evidenced by the structural frames depicting the preservation of beta-strands at multiple time intervals (Supplementary Figure S6). On the contrary, CathB2a peptide analogs displayed a reduced number of hydrogen bonds, diminished beta-sheet content, and increased variability in their secondary structure during the simulation (Figure 8B). The structural snapshots further substantiate these findings, indicating a more dynamic and less stable conformation for CathB2a peptide analogs over the course of the simulation (Supplementary Figure S8). The secondary structure plots elucidate these distinctions, revealing that CathB1a peptide analogs consistently form beta-sheets, whereas CathB2a peptide analogs demonstrate transient helical content with negligible beta-sheet formation (Supplementary Figures S7 and S9, respectively).

The research findings combined indicate that CathB1a peptide analogs possess a greater tendency for self-aggregation and amyloid fibril formation, potentially leading to toxic effects if supplied to patients with AD. Additional investigations are necessary to empirically confirm these computational results.

## 4. Discussion

AD is a multifactorial neurodegenerative disorder that is regarded by the World Health Organization (WHO) as one of the greatest healthcare challenges nowadays [1]. Despite extensive research endeavors, no treatment facilitating complete recovery presently exists, rendering AD a primary target of pharmaceutical development [1,48]. Peptides have emerged as versatile therapeutic agents for the treatment of various diseases, including AD, due to their selectivity, effectiveness, and capacity to target complex protein–protein interactions that small molecules frequently fail to address [11,49,50,51,52,53]. Advances in peptide engineering and modification have overcome limitations such as inadequate stability and bioavailability, paving the way for their application in neurological disorders [11]. Our group has recently investigated peptide analogs derived from Clusterin, another protein co-localized in amyloid plaques, and proposed their use as potential inhibitors of A*β* fibril formation [15]. Based on these findings, we performed a computational study to investigate if peptide-analogues that correspond to APRs of Cathepsin B, a protease co-localized in the amyloid plaques, can act as inhibitors of the A*β* oligomers that are nowadays considered as the most toxic state during the amyloidogenic process [8]. It was postulated that these APR-derived analogs would interfere with the pathogenic *β*-strand conformation, which is intimately linked to the transition of soluble A*β* helical monomers to toxic, insoluble oligomers [54]. By focusing on this critical structural motif, these peptide analogs present a viable approach to mitigate A*β* aggregation and its subsequent neurotoxicity. Each peptide analog was docked to the oligomer of A*β* and MD simulations were performed to evaluate its effect on the denaturation of the oligomer.

The results of the MD simulations indicate that the tested peptide analogs, CathB1, CathB1a, CathB2, and CathB2a, had an effect on the oligomeric state of A*β* by reducing the number of residues responsible for the formation of the *β*-sheet and the number of hydrogen bonds formed between *Aβ* monomers. These results are promising since the formation of *β*-sheets and hydrogen bonds are the key components of the A*β* oligomeric structure that subsequently form amyloid fibrils. Considering the pathological importance of *β*-strands in amyloidogenesis, reducing *β*-strand content is a crucial strategy for inhibiting A*β* aggregation.

Particularly, peptide analogs CathB1, CathB1a, and CathB2a demonstrated the best results regarding A*β* oligomer destabilization. CathB1 exhibited a significant destabilizing effect, decreasing *β*-strand content by 52.67% and inter-chain hydrogen bonds by 50.66%. This considerable disruption of the *β*-sheet-rich core was accompanied by conformational alterations toward helical structures, an increasing trend in structural deviations (RMSD plot), increased residue flexibility (RMSF analysis), and structural expansion of the oligomeric structure (Rg analysis), highlighting its ability to destabilize the A*β* oligomer architecture, a vital step toward inhibiting amyloid fibril formation. Regarding peptide analogs CathB1a and CathB2a, they both displayed substantial instability, diminishing the number of residues participating in *β*-strands by 47.92% and 44.91%, respectively, in addition to notable reductions in the number of hydrogen bonds formed between the chains of A*β* oligomer: 42.82% for CathB1a and 44.91% for CathB2a. Furthermore, both peptide analogs elicited significant structural transformations, as revealed by RMSD, RMSF, and Rg analyses, suggesting enhanced flexibility and a shift toward less compact conformations with the emergence of α-helices and 3_10_ helices in regions formerly dominated by *β*-strands. Nevertheless, the peptide analog CathB1a demonstrated a strong propensity for self-aggregation, as evidenced by MD self-assembly simulations. Its potential to generate stable *β*-sheet-rich aggregates raises apprehensions regarding potential toxicity if utilized as a therapeutic agent. Conversely, the peptide analog CathB2a exhibited lesser self-aggregation tendencies, integrating efficient destabilization with an improved safety profile. As far as peptide analog CathB2 is concerned, a more moderate destabilization was caused on the structure of the Aβ oligomer, with the *β*-strand content reduced by 33.61% and hydrogen bonds by 23.30%. CathB2, although less potent than the other peptide analogs, still disrupted localized *β*-sheet regions and increased residue flexibility, as shown by its RMSF and Rg profiles. These findings point to the fact that the peptide analog CathB2 may function as a less potent inhibitor compared to the other peptide analogs.

The umbrella sampling method further elucidated differences in binding affinities between the peptide analogs and the A*β* oligomer. Specifically, CathB1 exhibited weaker binding; however, its stabilizing effects remain considerable. On the contrary, CathB2′s lower binding affinity correlates with its comparatively milder impact on oligomer disruption. Peptide analogs CathB1a and CathB2a demonstrated the most robust binding interactions with the A*β* oligomer, reflected by free energy values of roughly −15 kcal/mol and −25 kcal/mol, respectively. These strong interactions suggest that both peptide analogs effectively bind to the oligomer and compromise its stability, possibly serving as promising peptide inhibitors of amyloid fibril formation. Notwithstanding its self-aggregation issues, CathB1a exhibited a high affinity for A*β* oligomer, whereas CathB2a presented a more equitable profile for binding strength and safety.

A critical consideration in peptide-based drug development that must be strongly considered is the potency of peptides that are predicted to correspond to APRs to self-assemble and form amyloid fibrils. In such a case, that peptide would be unsuitable for use as a potential drug due to its propensity to form aggregates that could be cytotoxic, and hence harmful for the patient [15,55]. The pronounced self-aggregation tendency of the peptide analog CathB1a, as observed by MD self-assembly simulations, reduces its viability as a potential peptide drug that can be administered to patients, albeit its significant destabilizing effects. On the other hand, the peptide analog CathB2a emerges as more advantageous choice, as it induces substantial oligomer disruption while maintaining a lower risk of forming toxic aggregates.

It is essential to emphasize that these results are derived from MD simulations, which offer vital mechanistic insights but necessitate additional experimental validation. Future in vitro and in vivo studies are required to verify the efficacy, stability, and safety of the proposed peptide analogs as therapeutic agents. Nonetheless, this study establishes a solid foundation for the rational design of peptide-based inhibitors targeting A*β* oligomers, and underscores the therapeutic potential of APR-derived peptide analogs of Cathepsin B in combating Alzheimer’s disease.

## 5. Conclusions

In summary, this study highlights the therapeutic potential of four peptide analogs derived from the APRs of Cathepsin B as inhibitors of A*β* oligomers, a pivotal target in AD. According to the results of the MD simulations, it is revealed that the peptide analogs CathB1, CathB1a, and CathB2a were the most proficient in disrupting the A*β* oligomeric structure by reducing the number of residues assigned to *β*-strands and the number of hydrogen bonds formed between the chains of A*β* oligomer, both of which constitute the pathological characteristics of amyloid aggregation.

Of the evaluated peptide analogs, CathB2a (^176^VYSDFLLY^183^) emerged as the most promising candidate, demonstrating an effective capacity to destabilize the A*β* oligomer while exhibiting low self-aggregation tendencies, thus addressing critical safety considerations for peptide-based therapeutics. The peptide analog CathB1a (^30^WAFGAV^35^), despite displaying potent destabilizing effects and a high binding affinity for A*β* oligomers, showed an apparent propensity for self-assembly and the subsequent possible formation of toxic aggregates, thereby diminishing its viability as a therapeutic agent.

These findings emphasize the importance of targeting *β*-strand conformations and inter-chain hydrogen bonding to inhibit A*β* oligomer stability, a key component in AD pathology. The present study provides an adequate framework for the development of peptide-based inhibitors aimed at A*β* oligomers, with the peptide analog CathB2a standing out as a potent candidate due to its balance of efficacy and safety, presenting a novel approach for therapeutic strategies against AD.

## Figures and Tables

**Table 1:** Peptide analogs that correspond to the APRs of Cathepsin B are presented with their code name and sequence length.

Peptide Name	Sequence	Length
CathB1	^28^SCWAFGAVEAISDRICIHTNA^48^	21
CathB2	^172^GAFSVYSDFLLYKSGVYQ^189^	18
CathB1a	^30^WAFGAV^35^	6
CathB2a	^176^VYSDFLLY^183^	8

**Table 2:** The average number of hydrogen bonds and residues assigned to *β*-strands in A*β* oligomer due to its interaction with each peptide analog during the first and last 10 ns and the respective percentage of decrease.

Peptide	First 10 ns		Last 10 ns		% Decrease Between First and Last 10 ns	
	Hydrogen Bonds	DSSP (*β*-Strands)	Hydrogen Bonds	DSSP (*β*-Strands)	Hydrogen Bonds	DSSP (*β*-Strands)
CathB1	62.77	73.00	30.97	34.55	−50.66	−52.67
CathB2	70.83	66.79	54.33	44.34	−23.30	−33.61
CathB1a	62.54	61.78	35.76	32.18	−42.82	−47.92
CathB2a	71.56	72.75	37.94	40.08	−46.98	−44.91

## Data Availability

The original contributions presented in this study are included in the article/Supplementary Materials. Further inquiries can be directed to the corresponding author.

## Supplementary Materials

The following supporting information can be downloaded at: https://www.mdpi.com/article/10.3390/biom15010028/s1, and the MD simulation files for the conducted analyses (MD.gro and MD.xtc) are accessible at: https://uoa2-my.sharepoint.com/:f:/g/personal/npapand_o365_uoa_gr/EogavetgbNtGvfhZHgmzTbUBYkrDeoJ5jK4PJrB8nUWpkw?e=EshpDi (accessed on 30 October 2024). These materials include the detailed molecular dynamics simulation commands and parameters used, along with supplementary figures: Figure S1: Simulation frames of the complex A*β*_42_ oligomer–CathB1 peptide analog at 0 ns, 100 ns, 200 ns, 300 ns, 400 ns, and 500 ns. Figure S2: Simulation frames of the complex A*β*_42_ oligomer–CathB2 peptide analog at 0 ns, 100 ns, 200 ns, 300 ns, 400 ns, and 500 ns. Figure S3: Simulation frames of the complex A*β*_42_ oligomer–CathB1a peptide analog at 0 ns, 100 ns, 200 ns, 300 ns, 400 ns, and 500 ns. Figure S4: Simulation frames of the complex A*β*_42_ oligomer–CathB2a peptide analog at 0 ns, 100 ns, 200 ns, 300 ns, 400 ns, and 500 ns. Figure S5: Radius of the gyration (Rg) profiles of A*β* oligomer complexes with CathB peptide-analogues over 500 ns molecular dynamics simulations. Figure S6: Simulation frames of the peptide analog CathB1a at 0 ns, 200 ns, 400 ns, 600 ns, 800 ns, and 1000 ns. Figure S7: Secondary structural analysis of the peptide analog CathB1a over the course of the simulation. Figure S8: Simulation frames of the peptide analog CathB2a at 0 ns, 200 ns, 400 ns, 600 ns, 800 ns, and 1000 ns. Figure S9: Secondary structural analysis of the peptide analog CathB1a over the course of the simulation.
