# Supplementary material for: Co-Localized in Amyloid Plaques Cathepsin B as a Source of Peptide Analogs Potential Drug Candidates for Alzheimer’s Disease
Source: Biomolecules. 2024 Dec 30;15(1):28. doi: 10.3390/biom15010028 (PMC11762647; doi:10.3390/biom15010028)
Supplement: Supplementary file 1 [file biomolecules-15-00028-s001.zip › biomolecules-3317856-supplementary.pdf]

Supplementary File

A protease co-localized in amyloid plaques as the source of peptide-analogues, drug candidates for Alzheimer's Disease

Marilena K. Theodoropoulou, Konstantina D. Vraila, Nikos C. Papandreou, Georgia I. Nasi and Vassiliki A. Iconomidou

1.1 Molecular Dynamics Simulation Commands

This section presents a comprehensive description of all terminal commands and script parameters employed in the molecular dynamics (MD) simulations conducted in this work. These commands ensure reproducibility and enable other researchers to replicate the computational studies.

Preprocessing Commands

```
# Step 1: Generate Topology

# Generate topology file for the system

gmx pdb2gmx -f input.pdb -o processed.gro -p topol.top -water tip3p

# Step 2: Define Simulation Box

# Define a cubic box with at least 1.2 nm distance between the protein and box
edges

gmx editconf -f processed.gro -o newbox.gro -c -d 1.2 -bt cubic


# Step 3: Solvate the System

# Add solvent molecules to the defined simulation box

gmx solvate -cp newbox.gro -cs spc216.gro -o solvated.gro -p topol.top


# Step 4: Add Ions

# Add ions to neutralize the system

gmx grompp -f ions.mdp -c solvated.gro -p topol.top -o ions.tpr

gmx genion -s ions.tpr -o solvated_ions.gro -p topol.top -pname NA -nname CL -
neutral
```

```
# Step 5: Energy Minimization

# Prepare the system for energy minimization

gmx grompp -f em.mdp -c solvated_ions.gro -p topol.top -o em.tpr

# Perform energy minimization

gmx mdrun -v -deffnm em


# Step 6: NVT Equilibration

# Prepare the system for NVT equilibration

gmx grompp -f nvt.mdp -c em.gro -p topol.top -o nvt.tpr

# Run NVT equilibration

gmx mdrun -v -deffnm nvt


# Step 7: NPT Equilibration

# Prepare the system for NPT equilibration

gmx grompp -f npt.mdp -c nvt.gro -p topol.top -o npt.tpr

# Run NPT equilibration

gmx mdrun -v -deffnm npt
```

Simulation Commands

```
# Production MD Run

gmx grompp -f md.mdp -c npt.gro -p topol.top -o md.tpr

# The following script was executed on the ARIS computational platform:


#!/bin/bash

####################################

#     ARIS slurm script template   #

#                                  #

# Submit script: sbatch filename   #

#                                  #

####################################
```

```
#SBATCH --job-name=cathb1a_1_310  # Job name
#SBATCH --ntasks=200     # Number of processor cores (i.e. tasks)
#SBATCH --nodes=10     # Number of nodes requested
#SBATCH --ntasks-per-node=20     # Tasks per node
#SBATCH --cpus-per-task=1     # Threads per task
#SBATCH --time=48:00:00   # walltime
#SBATCH --partition=compute     # Partition(s)
#SBATCH --mem-per-cpu=1024M   # memory per CPU core
#SBATCH --account=pr007003_thin    # Accounting project

export I_MPI_FABRICS=shm:dapl

export OMP_NUM_THREADS=$SLURM_CPUS_PER_TASK

# LOAD MODULES
    module load gnu
    module load intel
    module load intelmpi
    module load  gromacs/2018.1

srun gmx_mpi mdrun -v -ntomp 1 -deffnm md
```

Re-Centering and Re-Wrapping Coordinates

```
# Re-center and re-wrap coordinates for trajectory analysis
gmx trjconv -s md.tpr -f full.xtc -o nojump.xtc -pbc nojump
gmx trjconv -s md.tpr -f nojump.xtc -o molcom.xtc -pbc mol -ur compact -
center
gmx trjconv -s md.tpr -f molcom.xtc -o fitted.xtc -fit rot+trans
gmx trjconv -s md.tpr -f fitted.xtc -o whole.xtc -pbc whole
```

Analysis Commands

```
# Extract Specific Frames from Trajectory
```

```
gmx trjconv -s md.tpr -f whole.xtc -b 0 -e 2 -o fisrt_frame.pdb
gmx trjconv -s md.tpr -f whole.xtc -b 99998 -e 100000 -o frame100ns.pdb
gmx trjconv -s md.tpr -f whole.xtc -b 199998 -e 200000 -o frame200ns.pdb
gmx trjconv -s md.tpr -f whole.xtc -b 299998 -e 300000 -o frame300ns.pdb
gmx trjconv -s md.tpr -f whole.xtc -b 399998 -e 400000 -o frame400ns.pdb
gmx trjconv -s md.tpr -f whole.xtc -b 499998 -e 500000 -o frame500ns.pdb

# Perform Secondary Structure Analysis Using DSSP
gmx do_dssp -s md.tpr -f whole.xtc -sc secondary_structure.xvg -o ss.xpm

# Calculate Root Mean Square Fluctuation (RMSF)
gmx rmsf -s md.tpr -f whole.xtc -o rmsf.xvg

# Calculate Root Mean Square Deviation (RMSD)
gmx rms -s md.tpr -f md.xtc -o rmsd.xvg -tu ns

# Calculate Hydrogen Bonds
gmx hbond -s md.tpr -f whole.xtc -num hbond.xvg -tu ns

# Calculate Radius of Gyration (Rg)
gmx gyrate -s md.tpr -f whole.xtc -o gyrate.xvg
```

Umbrella Sampling Commands

```
# Generate configurations for Umbrella Sampling
# Prepare pulling simulation to generate configurations
gmx grompp -f pull.mdp -c conf.gro -p topol.top -o pull.tpr
# Perform pulling simulation
gmx mdrun -v -deffnm pull

# Post-process umbrella sampling results
```

```
# Use WHAM (Weighted Histogram Analysis Method) for PMF calculations

gmx wham -it tpr-files.dat -if pullf-files.dat -o pmf.xvg -hist hist.xvg -unit
kCal
```

Additional Notes

All simulations were conducted using GROMACS version 2018.1.

The systems were solvated using the TIP3P water model.

The force field used was AMBER99SB-ILDN.

Periodic boundary conditions were applied in all directions.

Energy minimization used the steepest descents algorithm with a maximum of 2000 steps.

Temperature coupling was achieved with the Berendsen thermostat at 310 K.

Pressure coupling used the Berendsen barostat set to 1 atm.

Long-range electrostatic interactions were calculated using the Particle-Mesh Ewald method.

The LINCS algorithm was applied to constrain bond lengths.

This section ensures that the methodologies and tools used for the MD simulations are transparent and replicable.

1.2 Supplementary Figures

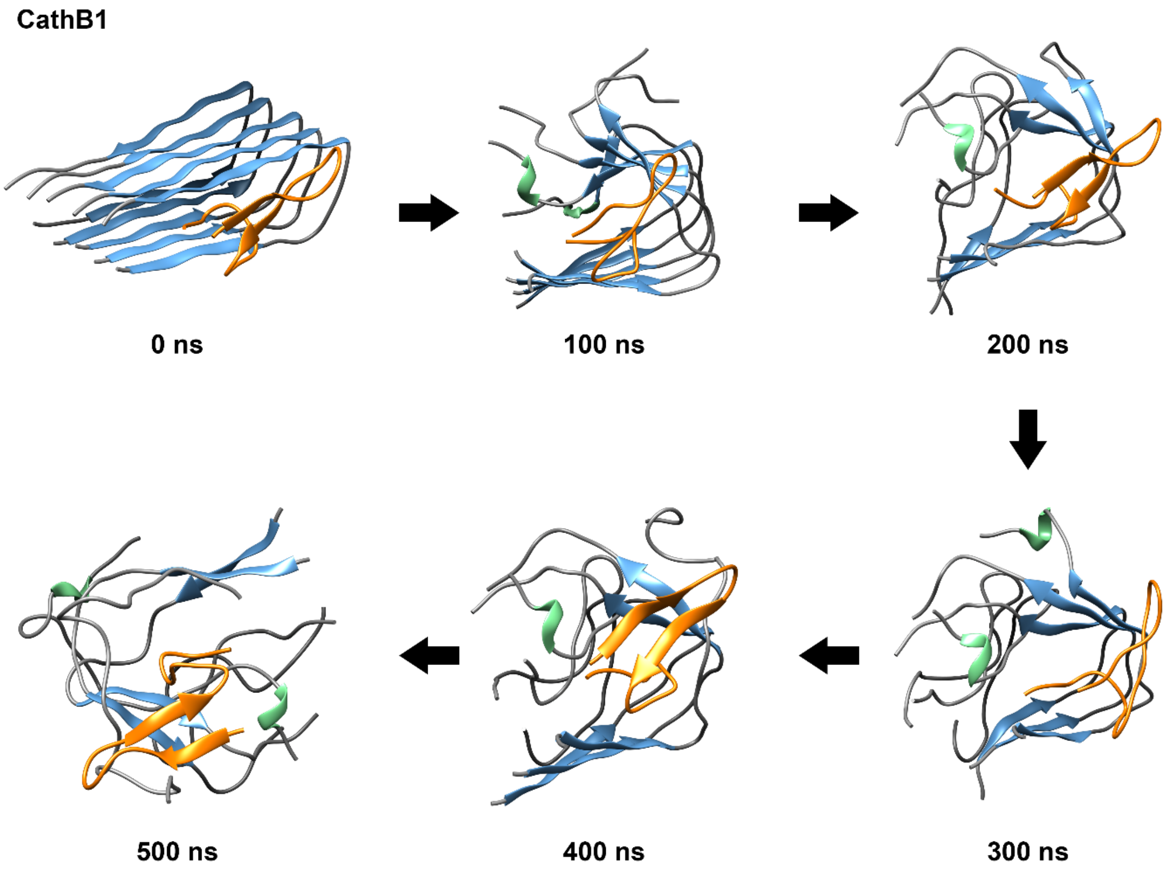

Figure S1. Simulation frames of the complex Aβ_{42} oligomer-CathB1 peptide-analogue at 0 ns, 100 ns, 200 ns, 300 ns, 400 ns and 500 ns. The coils of Aβ_{42} peptide are colored grey and CathB1 peptide-analogue is colored in orange. The secondary structural elements comprising the structure of the Aβ oligomer are represented by different colors. Beta-strands are colored blue and helices are represented by the color light green.

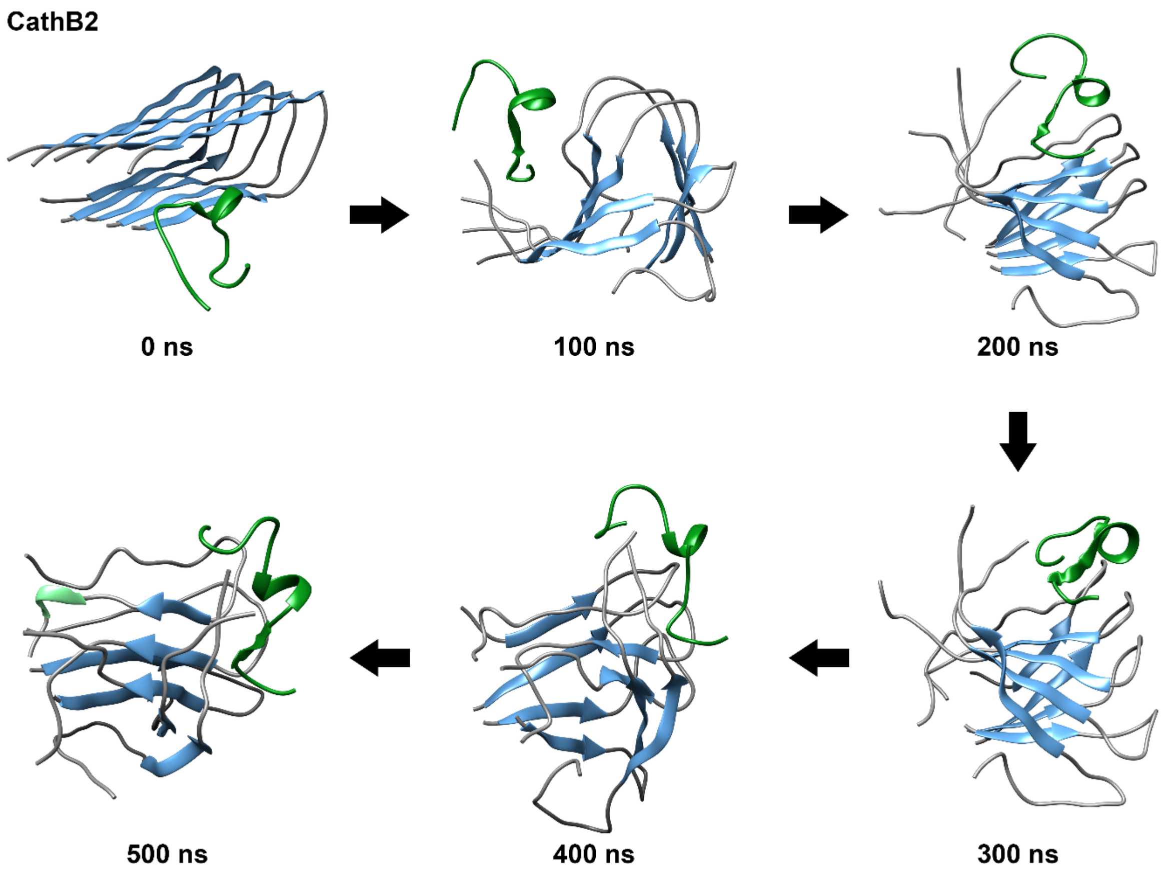

Figure S2. Simulation frames of the complex Aβ_{42} oligomer-CathB2 peptide-analogue at 0 ns, 100 ns, 200 ns, 300 ns, 400 ns and 500 ns. The coils of Aβ_{42} peptide are colored grey and CathB2 peptide-analogue is colored in dark green. The secondary structural elements comprising the structure of the Aβ oligomer are represented by different colors. Beta-strands are colored blue and helices are represented by the color light green.

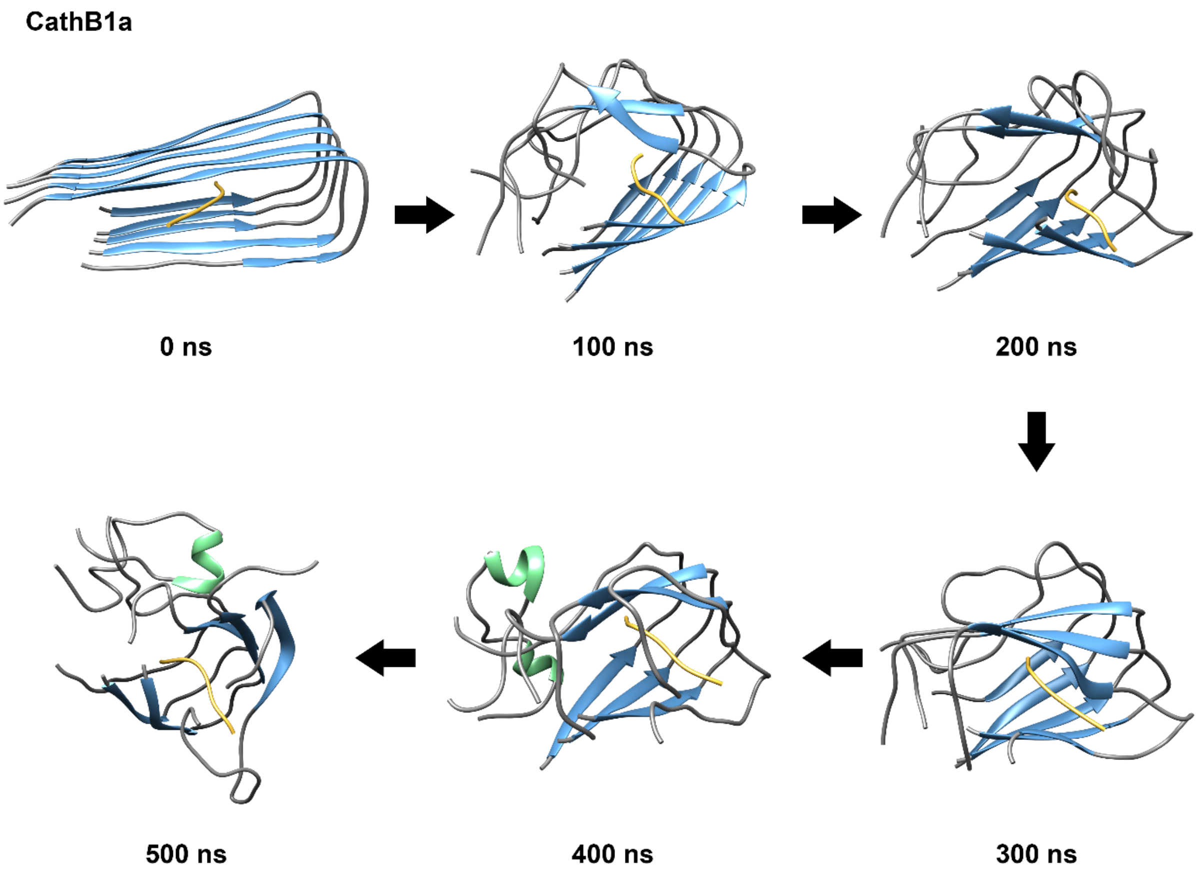

Figure S3. Simulation frames of the complex Aβ_{42} oligomer-CathB1a peptide-analogue at 0 ns, 100 ns, 200 ns, 300 ns, 400 ns and 500 ns. The coils of Aβ_{42} peptide are colored grey and CathB1a peptide-analogue is colored in yellow. The secondary structural elements comprising the structure of the Aβ oligomer are represented by different colors. Beta-strands are colored blue and helices are represented by the color light green.

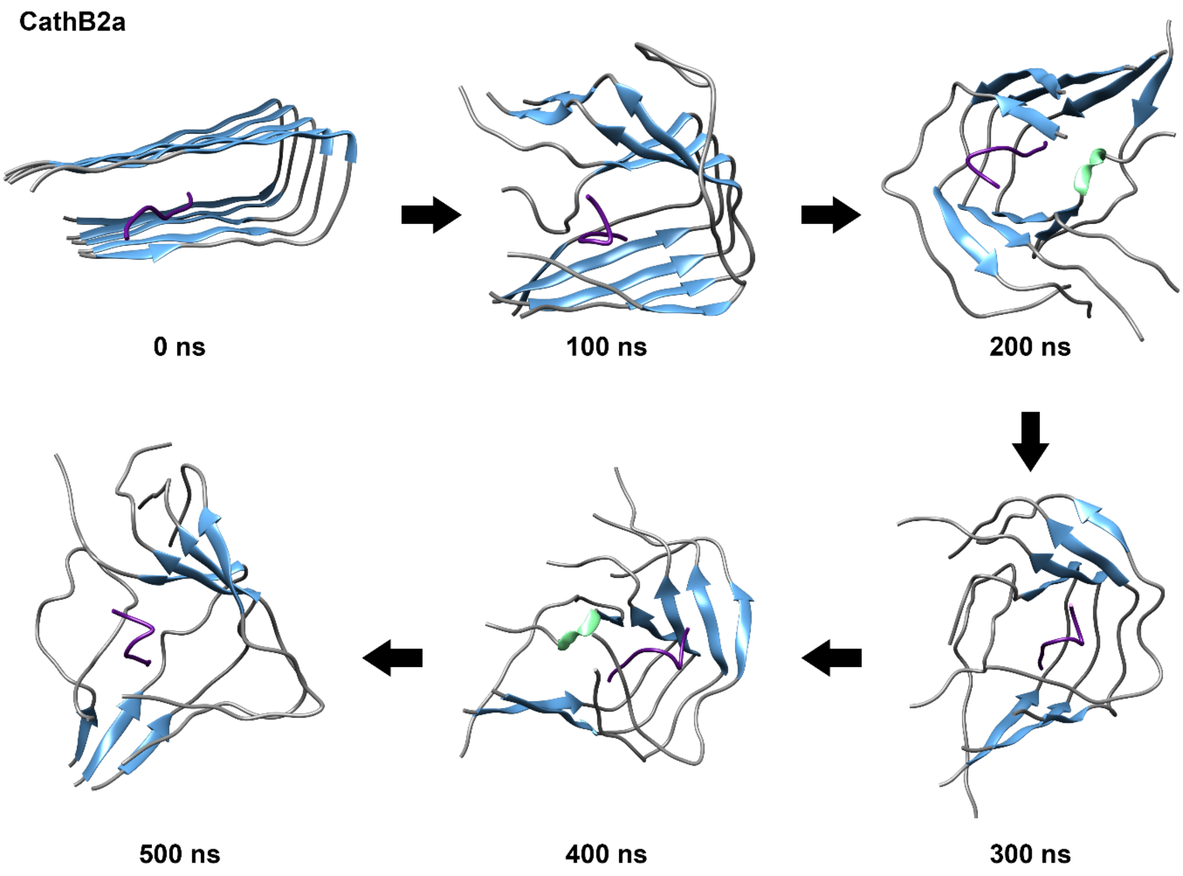

Figure S4. Simulation frames of the complex Aβ_{42} oligomer-CathB2a peptide-analogue at 0 ns, 100 ns, 200 ns, 300 ns, 400 ns and 500 ns. The coils of Aβ_{42} peptide are colored grey and CathB2a peptide-analogue is colored in purple. The secondary structural elements comprising the structure of the Aβ oligomer are represented by different colors. Beta-strands are colored blue and helices are represented by the color light green.

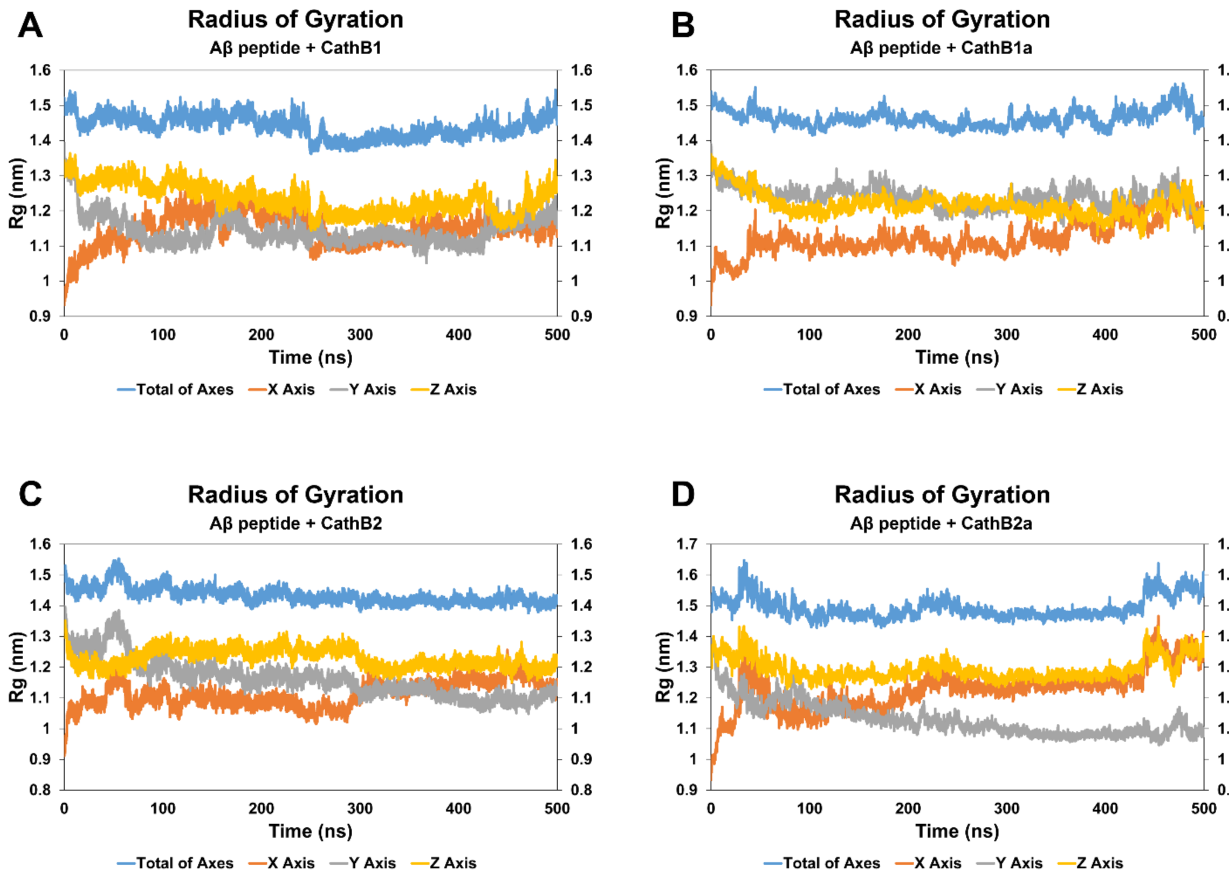

Figure S5. Radius of gyration (Rg) profiles of A*β* oligomer complexes with CathB peptide-analogues over 500 ns molecular dynamics simulations. (A) A*β* peptide + CathB1; (B) A*β* peptide + CathB1a; (C) A*β* peptide + CathB2; (D) A*β* peptide + CathB2a. The total Rg (blue) represents the overall compactness of the complexes, while Rg values along the X (orange), Y (gray), Z (yellow) axes indicate directional fluctuations.

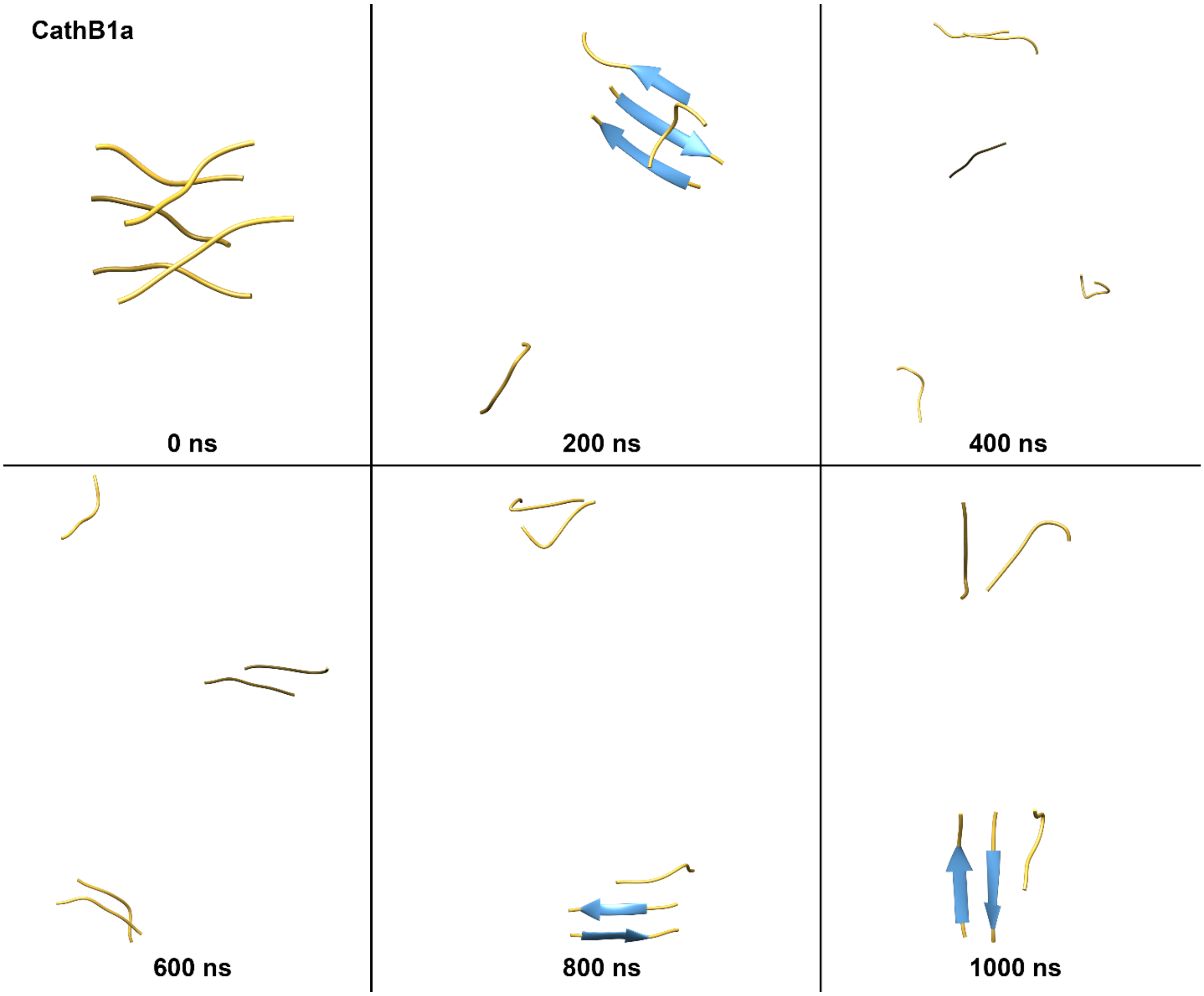

Figure S6. Simulation frames of the peptide-analogues CathB1a at 0 ns, 200 ns, 400 ns, 600 ns, 800 ns and 1000 ns. The chains of the peptides are colored yellow. The secondary structural element corresponding to beta-strands is represented by the color blue.

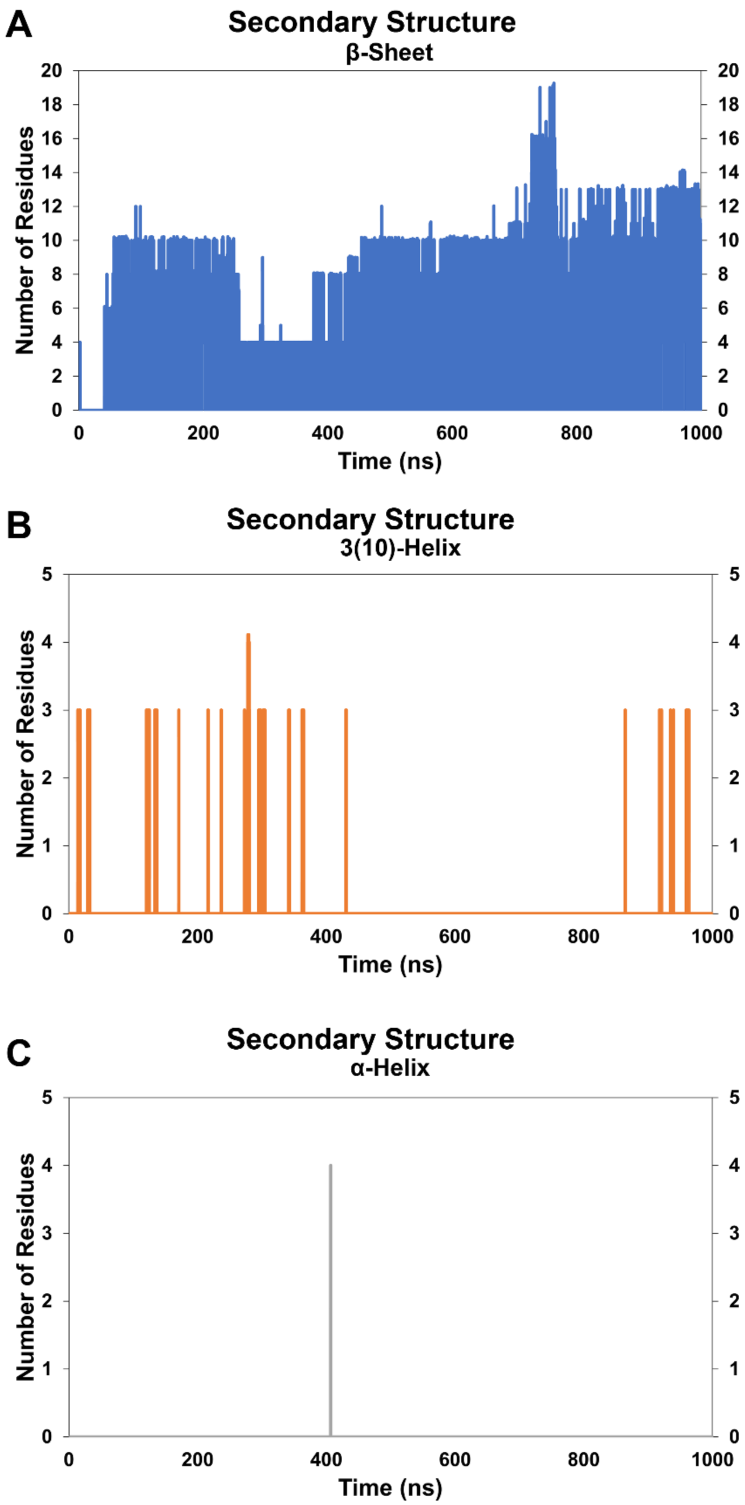

Figure S7. Secondary structural analysis of peptide-analogues CathB1a over the course of the simulation. (A) The number of residues adopting beta-sheet conformation is plotted against time. A gradual increase in beta-sheet content is observed after ~400 ns, with a significant rise toward the later stages of the simulation, indicating stable beta-sheet formation. (B) The number of residues forming 3_{10} helices is shown over time. Intermittent and transient 3_{10} content is observed primarily during the first half of the simulation.

(C) The number of residues adopting α-helix conformation remains negligible throughout the simulation, with a single transient occurrence around 400 ns.

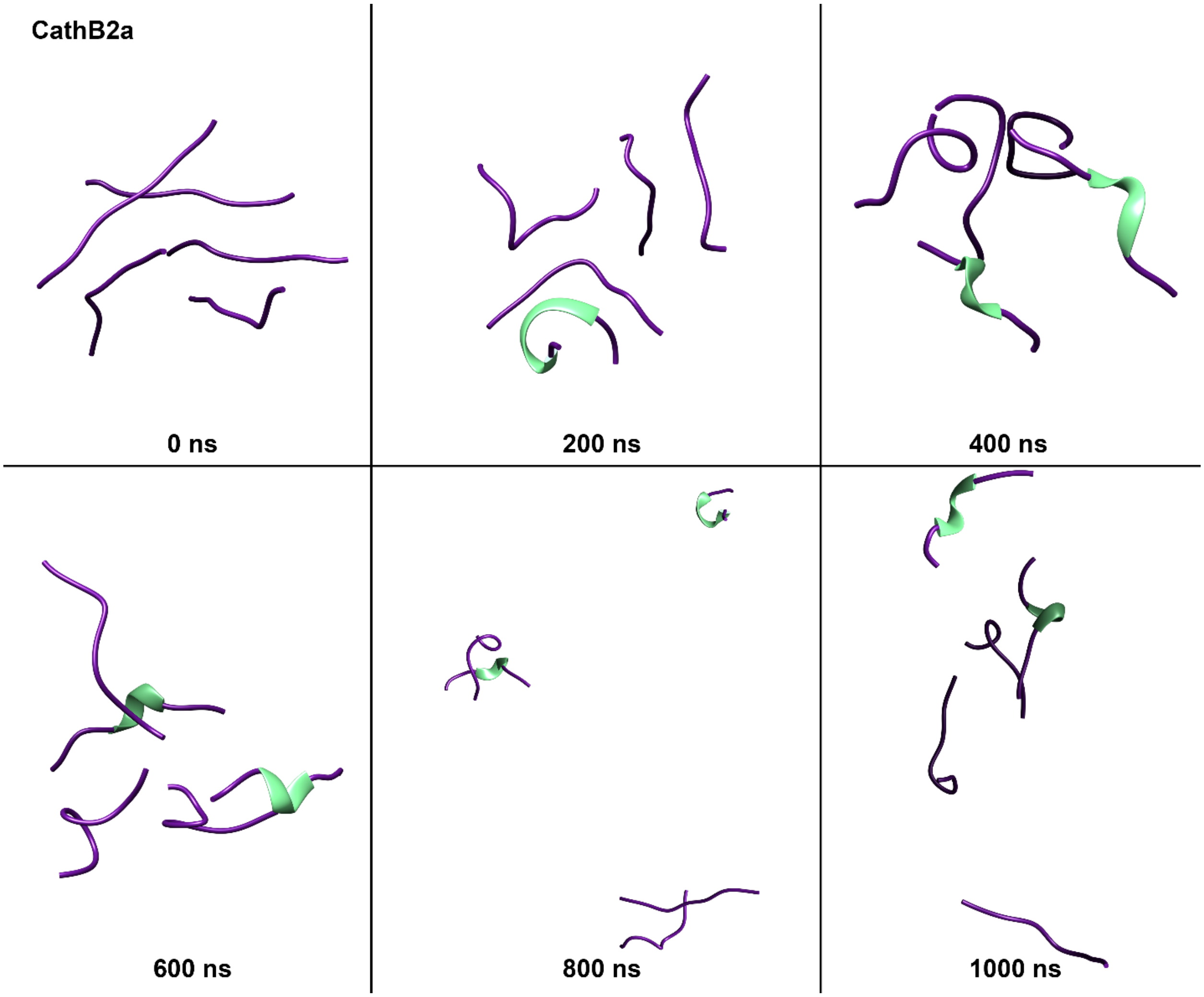

Figure S8. Simulation frames of the peptide-analogues CathB2a at 0 ns, 200 ns, 400 ns, 600 ns, 800 ns and 1000 ns. The chains of the peptides are colored purple. The secondary structural element corresponding to helices is represented by the color light green.

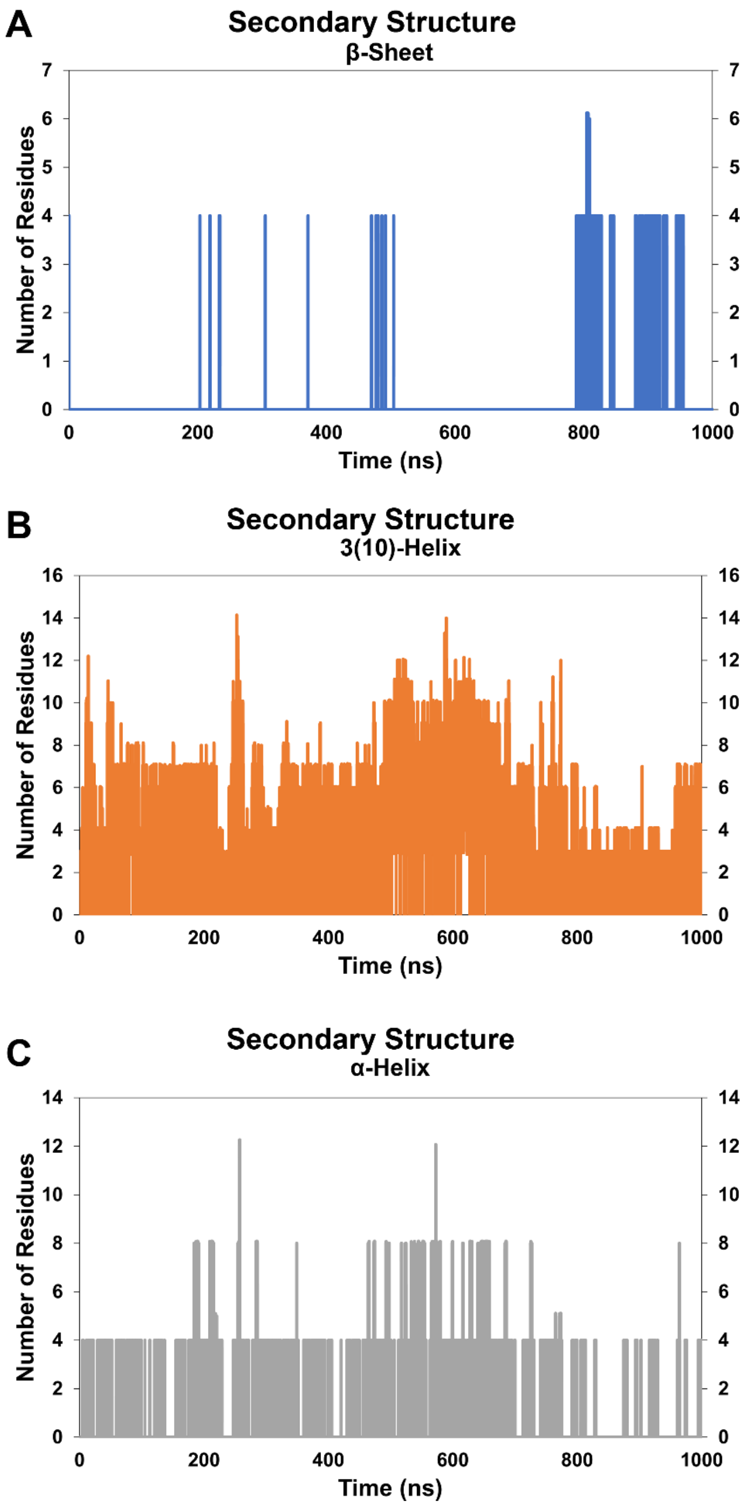

Figure S9. Secondary structural analysis of peptide-analogues CathB2a over the course of the simulation. (A) The number of residues adopting beta-sheet conformation remains sparse and intermittent throughout the simulation (0–1000 ns), with occasional peaks but no sustained beta-sheet formation. (B) The number of residues forming 3_{10} helices is plotted over time. A significant presence of 3_{10} helices is observed consistently across the simulation, suggesting a preference for transient helical structures. (C) The number

of residues adopting α-helix conformation fluctuates throughout the simulation, with multiple transient peaks. This indicates structural flexibility and a dynamic preference for helical conformations over beta-sheets.
